# Standardized MRI‐Based Quantification of Epicardial Adipose Tissue Volume: Reproducibility and Agreement With Cardiac Computed Tomography

**DOI:** 10.1002/jmri.70412

**Published:** 2026-06-23

**Authors:** Judith Gronwald, Svante S. Gersch, Zoé Böttiger, Clara Hagedorn, Torben Lange, Sören J. Backhaus, Sebastian Kelle, Constanze Schmidt, Karl Toischer, Andreas Schuster, Alexander Schulz

**Affiliations:** ^1^ Department of Cardiology and Pneumology University Medical Center Göttingen, Georg‐August University and German Center for Cardiovascular Research (DZHK), Partner Site Lower‐Saxony Göttingen Germany; ^2^ Department of Cardiology Campus Kerckhoff of the Justus‐Liebig‐University Giessen, Kerckhoff‐Heart‐Centre Bad Nauheim Germany; ^3^ Department of Cardiology and Angiology, Medical Clinic I University Hospital Giessen, Justus‐Liebig‐University Giessen Giessen Germany; ^4^ Cardio‐Pulmonary Institute (CPI) Giessen Germany; ^5^ Cardio‐Pulmonary Institute (CPI) Bad Nauheim Germany; ^6^ Department of Internal Medicine/Cardiology Charité Campus Virchow Clinic Berlin Germany; ^7^ German Center for Cardiovascular Research (DZHK) Partner Site Berlin Berlin Germany; ^8^ FORUM Medizin, Kardiologie Rosdorf Germany; ^9^ School of Biomedical Engineering and Imaging Sciences King's College London London UK

**Keywords:** cardiac CT, cardiac MRI, epicardial adipose tissue, standardization, validation

## Abstract

**Background:**

Epicardial adipose tissue volume (EATV) is increasingly recognized as a cardiometabolic risk marker associated with adverse outcomes. The most established approach for EATV quantification is cardiac computed tomography (CT). MRI offers a radiation‐free alternative allowing simultaneous assessment of myocardial function and tissue characteristics; however, standardization and validation are limited.

**Purpose:**

To evaluate a standardized MRI‐based method for EATV quantification and determine its agreement with CT.

**Study Type:**

Retrospective.

**Population:**

127 patients with aortic stenosis (AS) (78 ± 6 years; 38% female) who underwent paired CT and MRI and 11 volunteers (74 ± 7 years, 45% female) who underwent repeat MRI after ≥ 6 weeks.

**Field Strength/Sequence:**

Short‐axis balanced steady‐state free precession cine sequence at 3T (AS patients) and 1.5T (volunteers).

**Assessment:**

On CT, EATV was quantified by manual delineation of the visceral pericardium and voxel‐thresholding (−190 to −30 HU). MRI‐based EATV quantification used manual volumetry with delineation of the visceral pericardium and epicardium on end‐diastolic short‐axis cine stacks.

**Statistical Tests:**

Inter‐modality agreement was assessed by Spearman correlation and Bland–Altman analysis. Reproducibility was evaluated in 20 patients using intraclass correlation coefficient (ICC) and coefficient of variation (CoV). Scan‐rescan reproducibility for MRI‐derived EATV quantification was assessed using ICC and linear regression. *p* < 0.05 was considered significant.

**Results:**

Median EATV was significantly higher on MRI than CT (47 vs. 38 mL/m^2^), with a significant moderate correlation (*ρ* = 0.627) between measures. Inter‐ and intra‐observer analyses showed excellent reproducibility for both modalities (CT: intra‐observer ICC: 0.983, inter‐observer ICC: 0.994; MRI: intra‐observer ICC: 0.955, inter‐observer ICC: 0.970). MRI‐derived EATV quantification also showed excellent scan‐rescan reproducibility (ICC: 0.985).

**Data Conclusion:**

The standardized MRI‐based approach enabled highly reproducible EATV measurements with excellent repeatability. Agreement with CT was moderate, with systematically higher values on MRI, limiting direct comparability.

**Evidence Level:**

3.

**Stage of Technical Efficacy:**

2.

## Plain Language Summary

- Epicardial adipose tissue (EAT) is recognized as a marker of cardiometabolic risk. While it is usually measured using CT, MRI offers a radiation‐free alternative and can simultaneously assess tissue function and characteristics.
- This study evaluated an MRI‐based method for measuring EAT and compared it with CT. 127 patients undergoing CT and MRI and 11 volunteers undergoing repeat MRI were included.
- MRI measurements showed a moderate correlation with CT but consistently higher values. Importantly, the MRI method demonstrated excellent reproducibility between readers and across repeated scans.
- Because MRI and CT yield systematically different values, their measurements should not be used interchangeably.

## 1 Introduction

Epicardial adipose tissue (EAT) is a biologically active visceral fat depot in direct anatomical and biochemical continuity with the myocardium, lacking a separating fascia and sharing the same microcirculation [1]. This exposes the myocardium to adverse paracrine and vasocrine signaling from EAT, as well as to mechanical restrictive effects, thereby modulating cardiac metabolism [2], impairing myocardial function [3], and promoting inflammation and structural remodeling [4]. In line with these mechanistic considerations, EAT volume (EATV) has been associated with adverse effects across a range of cardiovascular diseases, including coronary artery disease [5], heart failure with preserved ejection fraction [6, 7], and atrial fibrillation [8]. Importantly, EATV can be modified by pharmacological therapy—most notably sodium‐glucose cotransporter‐2 inhibitors (SGLT2i) [9] and glucagon‐like peptide‐1 receptor agonists (GLP‐1RA) [10], further underscoring its potential as a therapeutic target.

To visualize and quantify EATV, several diagnostic modalities are currently available, primarily echocardiography, cardiac CT, and MRI, each with distinct strengths and limitations. While echocardiography enables rapid and cost‐effective measurement of EAT thickness, it does not allow volumetric quantification or assessment of spatial distribution [11]. CT is widely regarded as the reference standard due to its high spatial resolution and established three‐dimensional volumetric accuracy [12], including voxel‐wise quantification using Hounsfield unit thresholding, but its routine clinical use is limited by exposure to ionizing radiation. MRI may be a radiation‐free alternative, offering excellent soft‐tissue contrast and the potential for comprehensive EAT volumetry, alongside simultaneous assessment of myocardial function and tissue characteristics. For this reason, MRI is increasingly used as the modality of choice for studies aiming to quantify EATV [13, 14].

In particular, EAT volumetry derived from standard short‐axis cine acquisitions is practical, as it requires no additional sequences and also enables retrospective quantification. However, cine‐based EAT volumetry faces several inherent limitations like the choice of slice orientation [15], anisotropic resolution, or inter‐slice gaps that can result in incomplete spatial sampling or partial volume effects [16]. Even though delineation of the pericardium on cine images requires experience on the one hand, the dynamic assessment of the contractile cardiac cycle facilitates identification of the pericardial border and differentiation from pericardial effusion on the other hand [11]. Moreover, additional CMR techniques such as T1 mapping may assist in distinguishing EAT from adjacent fluid collections, including pericardial effusions [17]. Dedicated fat‐water separation techniques such as 3D‐Dixon imaging [18] significantly improved the discrimination between epicardial and pericardial fat compartments; however, these sequences require prospective acquisition as part of the imaging protocol and are not available by default [19].

Consequently, cine‐based approaches may provide a more accessible method for EATV quantification. However, systematic data on their reproducibility, repeatability, and accuracy compared with voxel‐wise CT‐based quantification remain limited. Such methodological validation is important to facilitate future robust risk stratification, clinical guidance, therapeutic decision‐making, and longitudinal monitoring.

Thus, the aim of this study was to systematically evaluate a standardized MRI‐based approach for EATV quantification with respect to image quality, reproducibility, and agreement with CT‐derived measurements.

## 2 Materials and Methods

### 2.1 Patient Cohort

Between 2017 and 2022, 135 patients with severe aortic valve stenosis were prospectively recruited to undergo paired CT and MRI prior to interventional transcatheter aortic valve replacement (TAVR), as part of an interdisciplinary research project (trial registration DRKS00024479) at the University Medical Center Göttingen, Germany. CT and MRI were performed within 1 week. Patients were excluded if they had severe coagulopathy, were legally incapacitated, or under guardianship. All patients provided written informed consent prior to participation; the study was approved by the local ethics committee (reference number 10/5/16). Furthermore, 11 volunteers were prospectively recruited to undergo repeated MRI examinations, separated by a minimum interval of 6 weeks at the Charité Campus Virchow‐Klinikum, Berlin, Germany, between 2017 and 2018 to assess scan‐rescan repeatability of EATV quantifications. Ethical approval for this study was obtained from the Ethics Committee of Charité—University Medicine Berlin (registration: EA4/112/16; German Clinical Trials Registry, DRKS, DRKS00015615). All participants provided written informed consent, and the study was conducted in accordance with the Declaration of Helsinki.

### 2.2 Cardiac Computed Tomography Imaging and Post Processing

Prospectively ECG‐triggered CT scans were performed on dual‐source multidetector scanners (Siemens SOMATOM Flash CT or Siemens SOMATOM Force CT, Siemens Healthcare, Erlangen, Germany), covering access path and device landing zone in preparation for the TAVR procedure [20]. A high‐pitch spiral acquisition mode with bolus tracking in the descending aorta was used for angiographic imaging. Contrast enhancement was achieved by injecting 80 mL of Imeron 350 (Bracco Imaging, Konstanz, Germany) or Ultravist 370 mg/mL (Bayer Vital GmbH, Leverkusen, Germany) at a flow rate of 4 mL/s followed by a 40 mL saline chaser with the same flow rate. Scan parameters were: 2 × 192 × 0.6 mm collimation, 250 ms rotation time, pitch of 3.2, automated tube current adaption. For subsequent analysis, a dedicated small field‐of‐view dataset centred on the heart was reconstructed with a medium‐soft convolution kernel (Siemens Bv36) and an isotropic resolution of 0.75–1.0 mm^3^.

### 2.3 MRI and Post Processing

MRI in the cohort scheduled for TAVR was performed on a 3.0T scanner (MAGNETOM Skyra, Siemens Healthcare, Erlangen, Germany) using a 32‐channel cardiac surface receiver coil. Breath‐held retrospectively gated bSSFP cine images were acquired in contiguous short‐axis (SAX) slices covering the whole ventricle for assessment of myocardial function and EATV quantification. Imaging parameters were: 25 reconstructed frames per cardiac cycle, echo time (TE) 1.5 ms, flip angle 55°, temporal resolution 55 ms, repetition time (TR) 3.5 ms, 16 phase‐encoding lines acquired per phase per cardiac cycle, slice thickness 7 mm, in‐plane spatial resolution 1.3 × 1.3 mm, and GRAPPA acceleration factor 3.

The volunteers were studied on a 1.5T scanner (Achieva, Philips Healthcare, Best, the Netherlands) using a five‐element phased‐array cardiac coil. bSSFP short‐axis cine acquisitions at both study time points had the following imaging parameters: 50 reconstructed frames per cardiac cycle (retrospectively gated), TE 1.6 ms, flip angle 60°, temporal resolution 35 ms, TR 3.3 ms, 11 phase‐encoding lines acquired per phase per cardiac cycle, slice thickness 8.0 mm, in‐plane spatial resolution 1.8 × 1.7 mm, and SENSE acceleration factor 2.

For both cohorts, ventricular volumetric analysis was performed by manual delineation of the endocardial and epicardial borders on contiguous short‐axis cine images covering the ventricles from base to apex. End‐diastolic volume, end‐systolic volume, stroke volume, and myocardial mass were quantified and indexed to body surface area. Ventricular ejection fraction was calculated as the ratio of stroke volume to end‐diastolic volume and expressed as a percentage [21].

### 2.4 Quantification of Epicardial Adipose Tissue Volume

**FIGURE 1:**
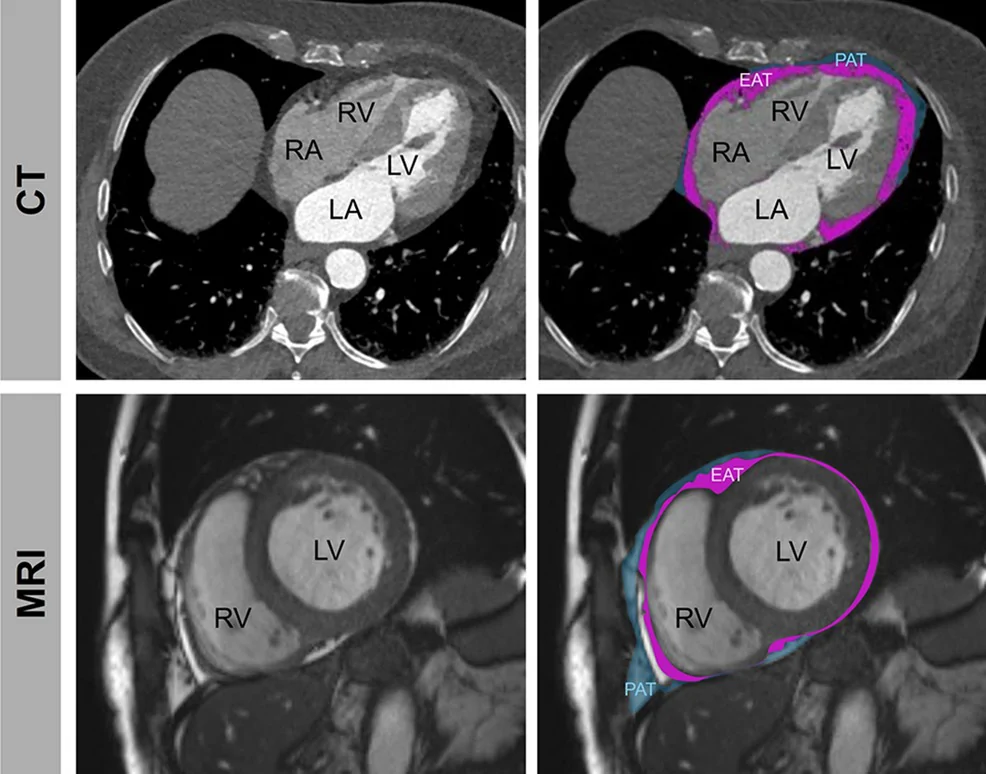
Representative visualization of epicardial adipose tissue (EAT) highlighted in pink and surrounding pericardial adipose tissue (PAT) highlighted in blue in both cardiac computed tomography (CT) and cardiac magnetic resonance imaging (MRI). LA, Left atrium; LV, Left ventricle; RA, Right atrium; RV, Right ventricle.

As illustrated in Figure 1, EAT was defined as adipose tissue located between the myocardium and the pericardium, whereas adipose tissue external to the pericardium was classified as pericardial adipose tissue. In the absence of established standards for CMR‐based EAT quantification, EATV was quantified using routinely acquired cardiac MRI sequences and the corresponding field of view, providing a pragmatic approach that allows comparison with previously reported EAT values. CT‐derived EAT quantification was performed in accordance with an established methodology [22].

### 2.5 Quality Assessment of EATV Quantification

**FIGURE 2:**
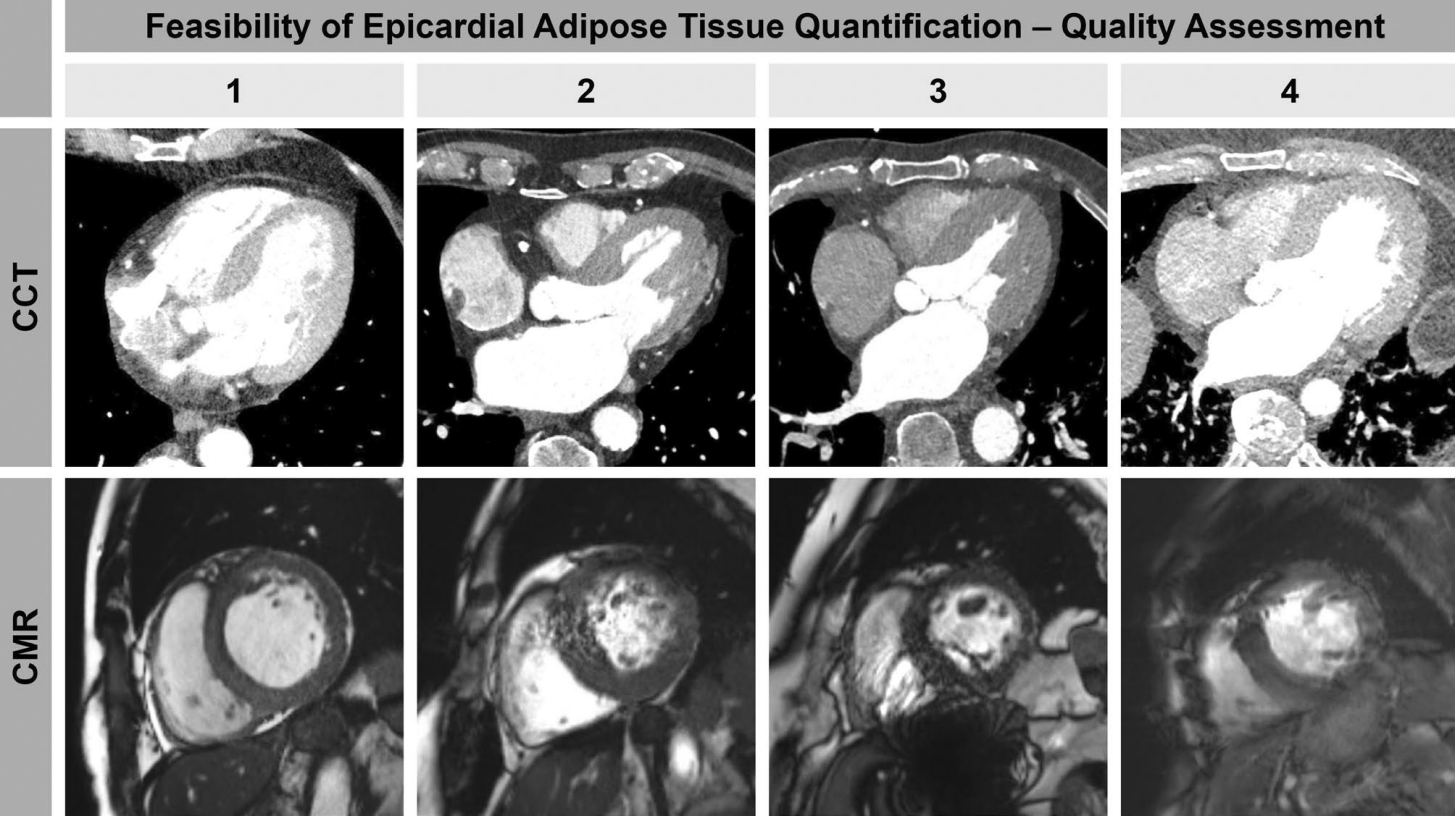
Representative cardiac computed tomography (CT) and MR images illustrating image quality scores 1–4 for the feasibility of epicardial adipose tissue (EAT) border delineation. Image quality was graded as score 4 (good), 3 (adequate), 2 (suboptimal), and 1 (poor) according to predefined criteria. CT images were windowed to optimize contrast for identification of the pericardial boundary. The images shown are representative of the respective quality categories. However, image quality scoring within the study was based on all slices used for EAT quantification in the respective modality, and the final image quality score was calculated as the mean across these slices.

Systematic assessment of image quality for delineation of EAT was performed for both CT and MRI scans using a four‐point Likert scale (MRI: observer 1 (J.G., reference observer) 3 years, observer 2 (S.G.) 2 years, observer 3 (A.S.) 11 years of experience; CT: observer 1 (J.G., reference observer) 1 year, observer 2 (S.G.) 1 year, observer 3 (A.S.) 4 years of experience). Table 1 provides an overview of the criteria applied to evaluate image quality and Figure 2 illustrates example ratings for each quality score for both CT‐ and MRI‐based EAT border delineation. In brief, a score of 4 indicated good, 3 adequate, 2 suboptimal, and 1 poor image quality.

**TABLE 1:** Four‐point Likert scale for image quality assessment.

Modality	Score	Evaluation	Criteria
CT	4	Good	The pericardial border is clearly visualized and can be manually delineated with high confidence along the entire heart
	3	Adequate	Small artifacts or isolated areas of low contrast but pericardial border remains well identifiable, no significant impact on manual delineation or automated fat detection
	2	Suboptimal	Less than 50% of the pericardial border is unclear, with localized areas of low contrast or artifacts
	1	Poor	More than 50% of the pericardial border is poorly defined or not reliably visible
MRI	4	Good	Epicardial and pericardial borders are clearly delineable. Differentiation between epicardial and pericardial fat is possible without limitations. No artifacts or blurring
	3	Adequate	Minor artifacts (e.g., minimal blurring, slight motion) or small areas with locally reduced contrast, borders are still well visible and traceable, no relevant impact on the accuracy of segmentation
	2	Suboptimal	Epicardial and/or pericardial borders are partially difficult to distinguish, due to blurring, motion/breathing artifacts or low contrast in less than 50% of pericardial borders delineated. Uncertainties on border delineation exist for less than 50% of myocardial circumference required
	1	Poor	Borders are not identifiable or are unreliable across more than 50% of myocardial circumference required. Significant artifacts, blurring or contrast issues severely impair distinction between epicardium, pericardium and EAT volume in more than 50% of pericardial borders delineated

Abbreviations: CT, cardiac computed tomography; EAT, epicardial adipose tissue.

### 2.6 Cardiac Computed Tomography‐Based EATV Quantification

CT‐based EATV quantification was performed by a single observer (Z.B., 1 year of experience) using dedicated software (Syngo.via, Siemens Healthineers AG, Forchheim, Germany; version: syngo CT VC28). In a first step, the pericardium was manually delineated on approximately 15 slices, from the main pulmonary artery bifurcation (cranial limit) to the last slice containing myocardial tissue (caudal limit). In a second step, contours on slices in between manual delineations were automatically interpolated and manually corrected if necessary to generate a three‐dimensional volume. EATV was automatically calculated as the total volume of voxels within a threshold of −190 to −30 HU [23] (Figure 1) and indexed to body surface area (BSA). Intra‐ and inter‐observer variability of CT‐based EATV quantification were assessed in 20 randomly selected patients: after a minimum period of three months, datasets were re‐evaluated by the same observer in a separate session, blinded to the initial measurements, and by a second independent and blinded observer (A.S., 4 years of experience).

### 2.7 MRI‐Based EATV Quantification

**FIGURE 3:**
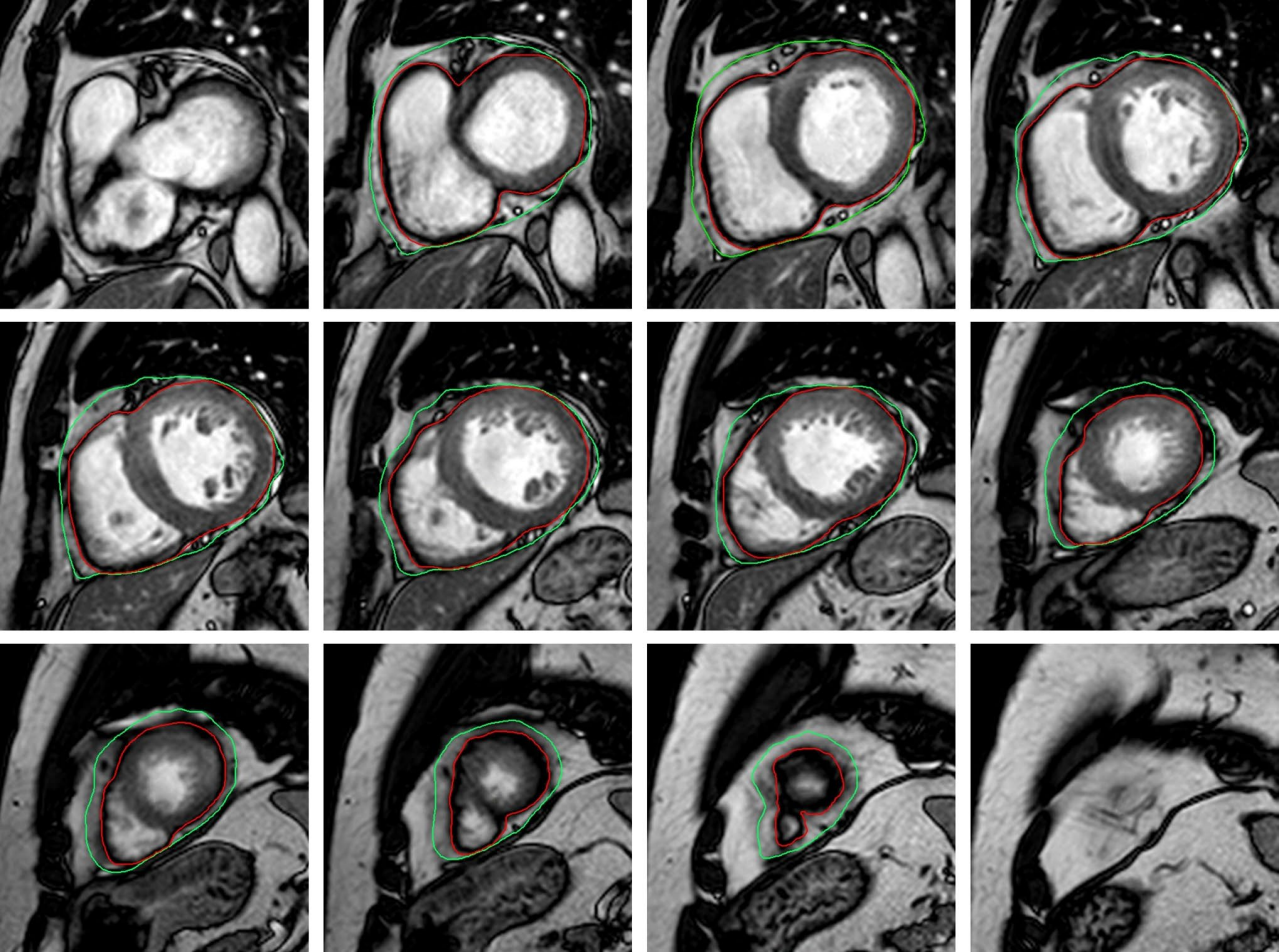
MRI‐derived quantification of epicardial adipose tissue using a short‐axis balanced steady‐state free precession cine sequence. The epicardial adipose tissue is located between the green line following the parietal layer of the serous pericardial border and the red line, which marks the epicardium (the visceral layer of the serous pericardium).

MRI‐based quantification of EATV was performed by one single observer (J.G., 3 years of experience) on end‐diastolic SAX slices using cvi42 (Circle Cardiovascular Imaging Inc., Calgary, Alberta, Canada). Epicardial and pericardial contours were manually delineated for each slice between the most basal slice showing a complete circumferential left ventricular (LV) myocardium, and the most apical slice in which the LV cavity was still visible (Figure 3). To assist pericardial contour delineation, dynamic information from cine images was reviewed to assess the motion of the myocardium and adipose tissue throughout the cardiac cycle. The total ventricular EATV was calculated using the modified Simpson's rule [15], the mass output by the software was divided by 1.05, corresponding to the density of fat tissue [15], and the result was indexed to BSA. In 20 randomly selected patients, intra‐ and inter‐observer variability were assessed by repeat blinded analysis by the same observer after a minimum interval of three months and by an independent second blinded observer (C.H., 1 year of experience). To assess scan‐rescan repeatability in the cohort of volunteers, the EATV quantification of the second MRI scan was performed blinded to the results of the first scan after a minimum time period of two months.

### 2.8 Statistical Analysis

Categorical variables are expressed as absolute numbers and percentages and compared using the *χ* ^2^ test. Continuous variables are reported as medians (with interquartile range) and analyzed using the nonparametric Mann–Whitney U test. Normality of continuous variables was assessed using the Shapiro–Wilk test and by visual inspection of histograms. Inter‐modality and inter‐observer agreement (pairwise comparisons with the reference observer) for the four‐point Likert scores was assessed using weighted Cohen's kappa (quadratic weights) and interpretation followed published guidelines [24]: *κ* < 0.00, poor; 0.00–0.20, slight; 0.21–0.40, fair; 0.41–0.60, moderate; 0.61–0.80, substantial; and 0.81–1.00, almost perfect agreement. Inter‐modality agreement of EATV quantification was also assessed using Spearman correlation and linear regression and visualized using Bland–Altman plots. Absolute values of Spearman's rank correlation coefficient (ρ) were interpreted as weak (< 0.5), moderate (≥ 0.5 to < 0.75), semi‐strong (≥ 0.75 to < 0.85), and strong (≥ 0.85) [25]. Furthermore, inter‐modality agreement was additionally evaluated in a predefined subgroup including only MRI and CT datasets rated as good or adequate (score 4 and 3) in image quality. Inter‐ and intra‐observer agreement was estimated using the intraclass correlation coefficient (ICC; two‐way mixed effects, absolute agreement, single measures) and coefficient of variation (CoV), as well as Cronbach's alpha, and visualized using Bland–Altman plots. Scan‐rescan repeatability was first assessed using the Spearman correlation coefficient, followed by an ICC analysis (two‐way mixed, absolute agreement, single measures) [26] and visualized using a linear regression. Furthermore, the coefficient of repeatability was calculated using the formula 1.96 × SD. A significance level of *p* < 0.05 was considered significant. All analyses were performed in SPSS version 30.0.0.0 (IBM, Armonk, New York, USA) and GraphPad Prism 10.6.1 (GraphPad Software, San Diego, CA, USA).

## 3 Results

### 3.1 Study Cohort and Feasibility of Epicardial Adipose Tissue Quantification

**FIGURE 4:**
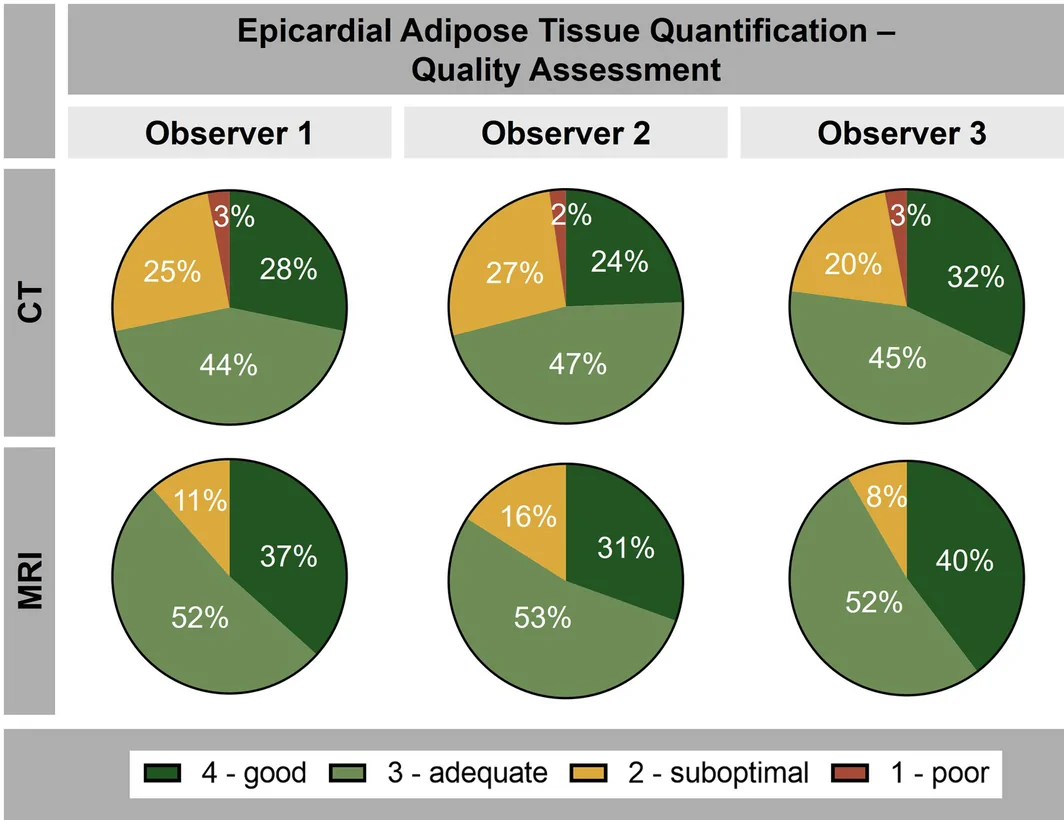
Comparison of image quality for cardiac computed tomography (CT)‐based and magnetic resonance imaging (MRI)‐based epicardial adipose tissue quantification.

Figure 4 provides an overview of the scores assigned by the three observers for each imaging modality. For CT‐based quantification, inter‐observer agreement was high, with weighted Cohen's *κ* values of 0.888 (95% CI: 0.812–0.963, *p* < 0.001) and 0.943 (95% CI: 0.904–0.981, *p* < 0.001) for the comparisons of observer 1 with observers 2 and 3, respectively. Similarly, MRI‐based assessments demonstrated comparable agreement, with weighted Cohen's *κ* values of 0.851 (95% CI: 0.744–0.958, *p* < 0.001) between observers 1 and 2 and 0.951 (95% CI: 0.911–0.991, *p* < 0.001) between observers 1 and 3. The subsequent analyses were based on the reference observer (1). 116 MRI scans (89%) were rated as good or adequate quality for EAT border delineation, 15 scans (11%) were considered suboptimal, and none were rated as poor; the median score was 3 (IQR: 1.0–2.0). For CT, 94 scans (72%) were rated as good (28%) or adequate (44%), 33 scans (25%) as suboptimal, and 4 scans (3%) as poor, with a median score of 3 (IQR: 1.5–3.0). In direct comparison, MRI scans were significantly more frequently rated as good or adequate than CT scans (116 (89%) vs. 94 (72%)). Agreement in scan quality between modalities at the patient level was poor, with a weighted Cohen's kappa (quadratic weights) of −0.013 (95% CI: −0.165 to 0.139; *p* = 0.868).

After excluding the four patients rated with poor CT image quality, the final study cohort comprised 127 patients (78 ± 6 years, 38% female) with paired CT and MRI data. Table 2 summarizes demographic and volumetric MRI assessments of the study cohort and the 11 volunteers.

**TABLE 2:** Cohort characteristics.

Parameter	TAVR cohort (*n* = 127)	Scan–Rescan cohort (*n* = 11)
Age, y	78 ± 6	74 ± 7
Sex female, *n* (%)	48 (38)	5 (45)
BMI (kg/m^2^)	28.1 ± 5.0	28.8 ± 4.3
LV Mi (g/m^2^)	80.5 (69.1–102.3)	56.9 (37.8–62.4)
LV EDVi (mL/m^2^)	78.5 (63.4–92.0)	92.9 (73.2–122.9)
LV ESVi (mL/m^2^)	30.1 (17.6–43.7)	42.4 (25.3–82.1)
LV SVi (mL/m^2^)	46.2 (37.5–54.0)	43.2 (33.1–50.1)
LV EF (%)	62.8 (48.3–73.6)	51.9 (33.2–60.0)
EATV (mL/m^2^)	46.5 (39.1–56.3)	13.8 (11.6–16.7)

Abbreviations: BMI, body mass index; EATV, epicardial adipose tissue volume; EDVi, end‐diastolic volume index; EF, ejection fraction; ESVi, end‐systolic volume index; LV, left ventricular; Mi, mass index; SVi, stroke volume index; TAVR, transcatheter aortic valve replacement.

### 3.2 Agreement of MRI‐Derived EATV With Cardiac Computed Tomography

Median EATV measured by CT was 38.0 mL/m^2^ (28.6–53.2) and was significantly lower than that measured by MRI (46.5 mL/m^2^ [39.1–56.3]). CT‐EATV and MRI‐EATV had a significant moderate positive correlation (*ρ* = 0.627, 95% CI: 0.507–0.724).

**FIGURE 5:**
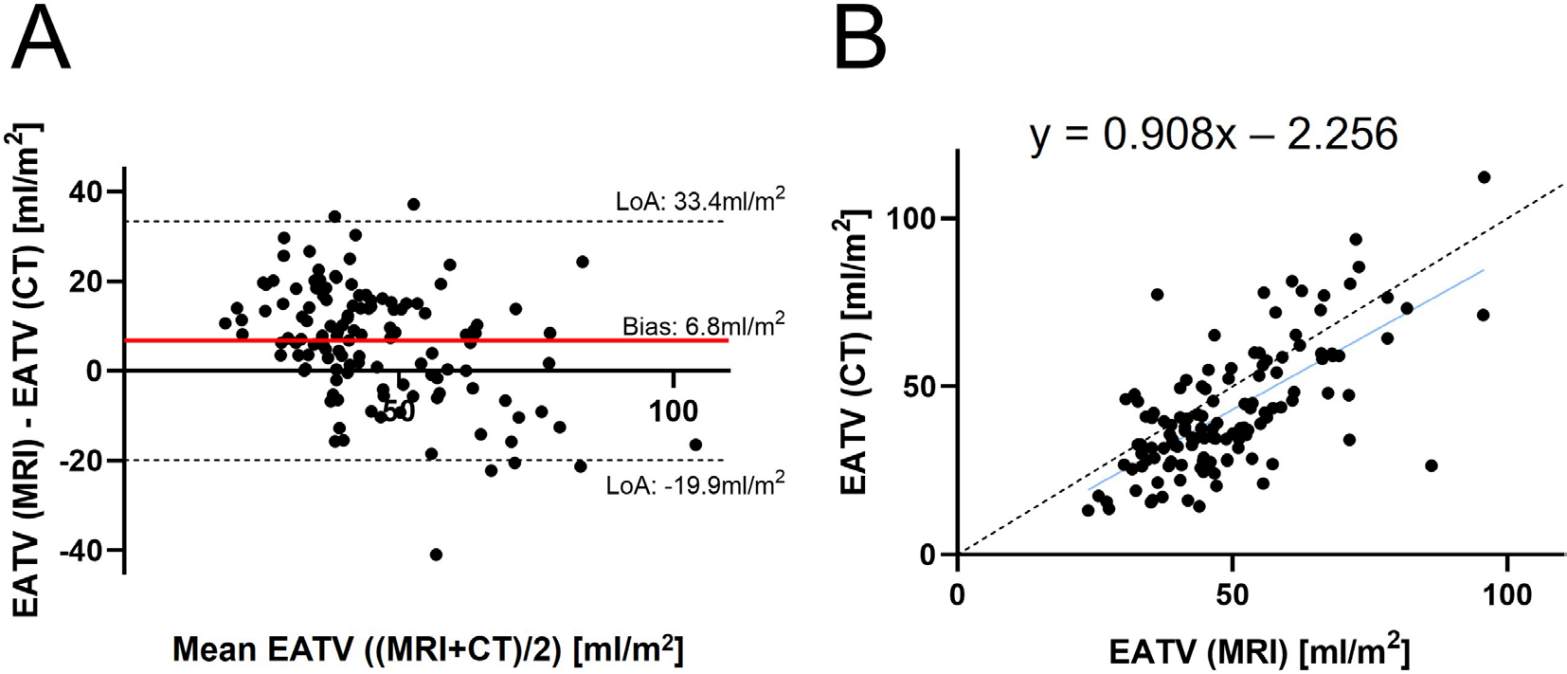
Bland Altman plot (A) and scatter plot with linear regression (B) of magnetic resonance imaging (MRI) and cardiac computed tomography (CT)‐derived measurements of epicardial adipose tissue volume (EATV, indexed to body surface area). (A) Bias 6.8 mL/m^2^, limits of agreement (LoA) −19.9 to 33.4 mL/m^2^; (B) *ρ* = 0.627, 95% CI: 0.507–0.724, *p* < 0.001.

MRI‐based EATV measurements had a mean bias of +6.8 mL/m^2^ (−19.9 to 33.4 mL/m^2^) compared with CT (Figure 5). For MRI‐based EATV quantification, a median of 10 (9–11) slices was analyzed per examination.

In a subgroup analysis including only patients with both CT and MRI scan quality rated as good or adequate for border delineation (score 4 or 3; *n* = 82), a significant moderate correlation was likewise observed (*ρ* = 0.686, 95% CI: 0.547–0.789).

### 3.3 Inter‐ and Intra‐Observer Variability

**FIGURE 6:**
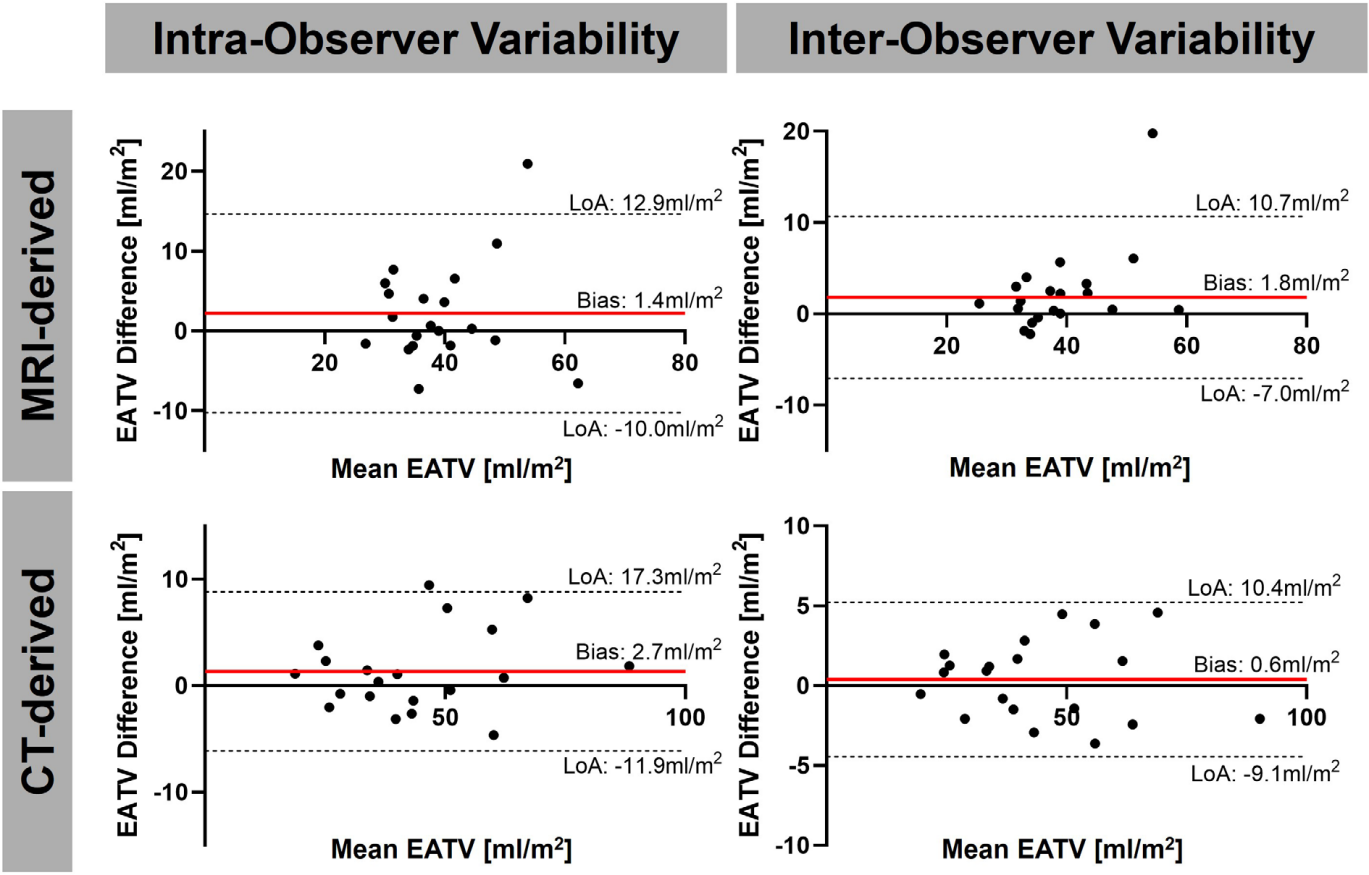
Intra‐ and inter‐observer variability of cardiac magnetic resonance (MRI)‐ and cardiac computed tomography (CT)‐based quantification of epicardial adipose tissue volume (EATV, indexed to body surface area) using Bland–Altman plots. LoA, Limit of agreement.

Results of inter‐ and intra‐observer analysis for EATV quantification using CT and MRI are presented in Table 3 and Figure 6. In the intra‐observer analysis, the ICC indicated excellent reproducibility for both CT (0.983, 95% CI: 0.956–0.993) and MRI (0.955, 95% CI: 0.891–0.982). Similarly, inter‐observer variability was excellent for both modalities (CT: ICC: 0.994 [95% CI: 0.984–0.998]; MRI: ICC: 0.970 [95% CI: 0.926–0.988]).

**TABLE 3:** Inter‐ and intra‐observer variability in CT‐ and MRI‐derived EATV measurements.

	CT	MRI
*Intra‐observer variability*		
ICC	0.983	0.955
95% CI	0.956–0.993	0.891–0.982
*p*	< 0.001	< 0.001
Cronbach's alpha	0.992	0.977
CoV [%]	8.6	8.7
Bland–Altman bias (mL/m^2^)	2.7	1.4
Bland–Altman limits of agreement (mL/m^2^)	−11.9 to 17.3	−10.0 to 12.9
*Inter‐observer variability*		
ICC	0.994	0.970
95% CI	0.984–0.998	0.926–0.988
*p*	< 0.001	< 0.001
Cronbach's alpha	0.997	0.985
CoV (%)	5.4	5.8
Bland–Altman bias (mL/m^2^)	0.6	1.8
Bland–Altman limits of agreement (mL/m^2^)	−9.1 to 10.4	−7.0 to 10.7

Abbreviations: CI, confidence interval; CoV, coefficient of Variation; CT, cardiac computed tomography; ICC, intraclass correlation coefficient; MRI, cardiac magnetic resonance imaging.

### 3.4 Scan‐Rescan Reproducibility of MRI‐Based EAT Volumetry

The second MRI scan was performed after a median time interval of 63 (50–87) days after the first scan.

**FIGURE 7:**
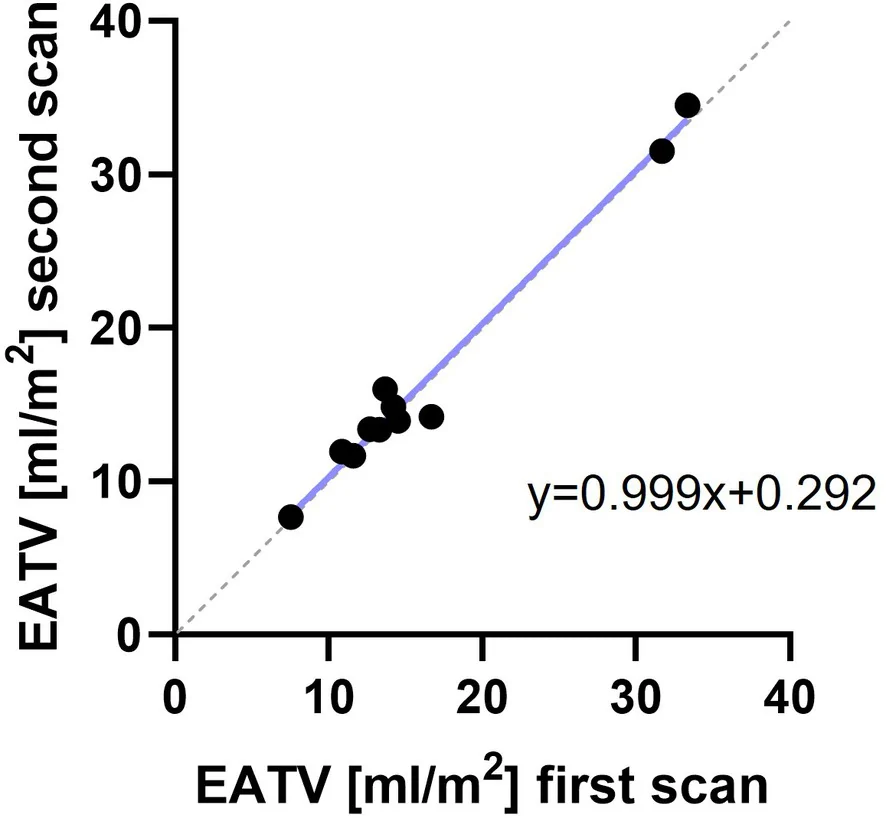
Linear regression demonstrating scan‐rescan repeatability for magnetic resonance imaging (MRI)‐derived epicardial adipose tissue volume (EATV) quantification in 11 volunteers; *ρ* = 0.900, 95% CI: 0.640–0.975, *p* < 0.001.

The mean EATV was 13.8 mL/m^2^ (11.6–16.7) in the first MRI scan and 13.9 mL/m^2^ (12.0–16.0) in the second scan. As illustrated in Figure 7, a linear relationship between measurements was observed (*ρ* = 0.900, 95% CI: 0.640–0.975; *y* = 0.999*x* + 0.292). EATV quantification in repeated scans had excellent repeatability as indicated by an ICC of 0.985 (95% CI: 0.949–0.996) with a Cronbach's alpha of 0.992. The coefficient of repeatability was 2.4 mL/m^2^.

## 4 Discussion

This study provides a comparison of standardized MRI‐based EATV quantification with CT‐based quantification. Overall, MRI‐based assessments were associated with higher image quality ratings for EATV evaluation; however, agreement at the individual patient level was low. EATV values derived from MRI were systematically higher than those obtained using CT, and there was only a moderate inter‐modality correlation. MRI showed excellent intra‐ and inter‐observer reproducibility for EATV quantification, comparable to CT‐based measurements, and further showed excellent scan‐rescan reproducibility across repeated MRI examinations.

Approaches to EATV quantification using MRI are heterogeneous, ranging from the choice of imaging plane (e.g., four‐chamber view [27] versus SAX [14]) to methodological details such as slice selection and the timing within the cardiac cycle. Although EATV can be quantified in both end‐diastolic [28] and end‐systolic [29] images, and end‐systolic imaging may offer advantages for delineation of the pericardial border in some individuals, the majority of published MRI studies have used end‐diastolic images [17, 30, 31]. Accordingly, EATV quantification in the present study was performed in end‐diastole to maximize comparability with the existing body of literature. While individual approaches may offer specific advantages and limitations, methodological standardization and validation are important to ensure comparability across studies.

This study compared image quality for EAT border delineation between MRI and CT using a structured quality assessment. MRI‐based delineation of EAT borders was predominantly rated as good or adequate and was overall superior to CT. The feasibility of MRI‐based EAT border delineation is further supported by prior studies, which reported low exclusion rates due to insufficient image quality when quantifying MRI‐derived EATV, ranging from 3% [32] and 4% [30] to 7% [33]. The high feasibility may be explained by the high soft‐tissue contrast of MRI and the use of cine imaging, which allows dynamic tracking of EAT boundaries throughout the cardiac cycle based on their relative motion with respect to the pericardium.

The present study demonstrated poor agreement at the patient level between CT‐ and MRI‐based image quality for EAT border delineation. This suggests that these image quality ratings may be driven by modality‐specific technical factors in individual cases rather than by patient‐related characteristics. The good image quality for MRI‐based EAT border delineation is further supported by the excellent intra‐ and inter‐observer reproducibility of MRI‐derived EATV quantification.

Despite the high image quality, a systematic bias toward higher EATV as measured by MRI was observed. This finding may appear counterintuitive, as the most basal portions of EATV at the level of the atria are not included in the MRI‐based method, whereas they are accounted for in the CT‐based quantification approach. Notably, a subgroup analysis restricted to patients with good or adequate scan quality for border delineation in both modalities demonstrated a similarly moderate inter‐modality agreement of absolute EATV values, supporting the assumption that this bias is primarily technical rather than attributable to image quality limitations. When comparing the two methods, it should be considered that CT‐ and MRI‐based EATV quantification is typically performed on different image orientations and different phases of the cardiac cycle, as well as in different respiratory phases [34]. The systematic overestimation of EATV by MRI compared to CT may be partly attributable to the larger slice thickness used for MRI acquisition. In our study, EATV quantification was performed on 8 mm MRI slices, whereas CT provides substantially thinner slices and higher spatial resolution. The resulting lower through‐plane resolution of MRI increases the likelihood of partial volume effects, which have previously been described when comparing MRI and CT measurements in other contexts [35]. Voxels at tissue interfaces may therefore contain a mixture of adipose and nonadipose tissue, which may lead to inclusion of non‐EAT voxels within the segmented volume and thus overestimation of EATV. The significantly higher EATV in MRI may also partly reflect differences in image acquisition and segmentation between MRI and CT, which may affect the delineation of EAT in anatomical regions where EAT is typically more pronounced (e.g., the interventricular grooves [36]). While such modality‐specific acquisition standards, as established in the literature [23, 37, 38, 39], introduce a potential source of bias related to the method of quantification rather than the imaging modality per se, they nonetheless enable consistent benchmarking and facilitate comparison with previously published EATV values across studies.

In addition, the two methods prescribe different slices thicknesses and spatial resolution. Consequently, the moderate correlation between MRI and CT likely reflects inherent methodological differences rather than limited measurement robustness. However, this finding should be considered in clinical practice and EATV measurements should be interpreted in the context of the imaging modality and approach to quantification used. A prior study based on a cohort of 92 individuals has compared CT‐ and MRI‐based EATV quantification using 3D Dixon water‐fat‐separated late gadolinium enhancement imaging (LGE‐Dixon) [40]. In contrast to our findings, the authors reported larger CT‐derived than MRI‐derived EATV values, and the study furthermore demonstrated superior agreement between both modalities. These discrepant results may, at least in part, be explained by methodological differences, as MRI analysis was performed in a CT‐equivalent image plane using a transverse stack of water‐ and fat‐separated images obtained from the 3D Dixon acquisition. While Dixon‐based techniques offer excellent fat‐tissue contrast [18] and hold promise for more precise delineation, their availability remains limited and they require an additional acquisition. A direct comparison of such approaches with established methods, particularly with respect to accuracy, remains an important objective for future studies.

Comparisons with previous studies [30, 31] using similar acquisition techniques are challenging, as even within a single imaging modality, absolute EATV shows considerable variability across reported patient cohorts and studies, attributable to multiple factors: First, inherent differences in patient characteristics and underlying disease states contribute to this variability; for example, MRI‐based EATV in a cohort of asymptomatic obese individuals was reported to be 41 mL/m^2^ [30], whereas in a cohort of patients with coronary artery disease, the median value was 60 mL/m^2^ [31]. Second, published CT‐derived EATV is frequently reported without indexing to BSA, limiting comparability across individuals and particularly between sexes; a median of 47 mL has been reported in a cohort with heart failure with preserved ejection fraction [39] and 63 mL in a different cohort that also had severe aortic valve stenosis [41] (for comparability: the median nonindexed EATV in our study cohort was 73 mL). Lastly, in addition to disease‐ and cohort‐specific differences, technical factors can also substantially contribute to variability in EATV measurements. For instance, a study exploring CT‐derived EATV assessment differences using different post‐processing software options showed median values of 53 mL for a freeware option, 93 mL for research software, and 157 mL using coronary software in a single cohort [42].

Beyond absolute differences in EATV, measurement reproducibility is an important determinant for the implementation of EATV quantification in clinical practice, particularly for longitudinal studies and therapy monitoring. Both CT and MRI methods of EATV quantification showed excellent intra‐ and inter‐observer variability. When comparing these findings to previous studies, there is again a wide range of results. While one study on CT‐based EATV reported excellent intra‐ and inter‐observer variability (ICC of 0.98 for both) [43], the study comparing different software for quantification found an ICC of 0.98 for the research, 0.75 for the coronary, and 0.73 for the freeware options [42]. A previous MRI‐based approach has also demonstrated strong agreement, with intra‐observer and inter‐observer ICCs of 0.86 and 0.81, respectively [30], suggesting that the use of a standardized methodology, as in the present study, may contribute to improved repeatability. Moreover, standardized CMR reader training for EATV quantification may further improve inter‐observer variability. Karamitsos et al. demonstrated this phenomenon in the context of ejection fraction assessment, when showing that a structured 2‐month training program significantly reduced operator‐induced variability in MRI contour delineation, with ejection fraction variability decreasing from 7.2% to 3.7% [44]. Although training effects were not specifically investigated in our study, readers with greater CMR experience rated the image quality for EAT border delineation more favorably than less experienced readers. However, quantitative EATV measurements demonstrated good reproducibility across readers overall, emphasizing the robustness of a standardized evaluation approach.

Finally, the proposed MRI‐based technique demonstrated excellent scan‐rescan reproducibility, which may facilitate translation [45, 46] into longitudinal assessments and therapy monitoring. Although the volumetric quantification does not provide qualitative information on adipose tissue composition, it is straightforward in its application and could be incorporated into routine clinical MRI assessments. For both clinical implementation and scientific applications, including multicentric studies, methodological harmonization and standardization are therefore essential. First automated MRI‐based EAT quantification approaches have already been proposed [46], which could further facilitate adoption; however, additional validation is required. To advance the potential of EATV as a cardiometabolic biomarker, subsequent steps should include the establishment of population‐specific reference values and clinically relevant cut‐off thresholds for therapeutic decision‐making and longitudinal monitoring.

## 5 Limitations

Patients with severe aortic valve stenosis represent a selected cohort characterized by advanced age and potential scanning challenges related to cardiac decompensation, limiting the generalizability of the findings. Nevertheless, the good agreement observed even in this more demanding cohort demonstrates the method's applicability in routine clinical practice. A further potential limitation is that observers received prior training from a single experienced reader, which may have introduced bias in the inter‐observer comparison. Lastly, scan‐rescan reproducibility was assessed on a 1.5T scanner, in contrast to the other analyses, which were performed on a 3.0T system due to the retrospective nature of the data analysis.

## 6 Conclusion

Using a standardized MRI‐based approach, EATV may be quantified with high reproducibility on routinely acquired cine short‐axis images. Compared with CT, MRI significantly overestimates EATV, which requires consideration for direct comparison across imaging modalities and studies.

## Funding

This study was supported by the German Research Foundation [DFG, CRC 1002, D1]. Alexander Schulz is funded by the German Research Foundation (Walter Benjamin Fellowship) and Svante Gersch is funded by the University Medical Center Göttingen—UMG Clinician Scientist Programme.

## Ethics Statement

The study was conducted in accordance with the Declaration of Helsinki and approved by the local ethics committee (reference number 10/5/16).

## Consent

All patients provided written informed consent prior to participation.

## Conflicts of Interest

The authors declare no conflicts of interest.

## Data Availability

The data underlying this article will be shared on reasonable request to the corresponding author.
